# A Cross-Species Systems Genetics Analysis Links APBB1IP as a Candidate for Schizophrenia and Prepulse Inhibition

**DOI:** 10.3389/fnbeh.2019.00266

**Published:** 2019-12-10

**Authors:** David G. Ashbrook, Stephanie Cahill, Reinmar Hager

**Affiliations:** ^1^Department of Genetics, Genomics and Informatics, University of Tennessee Health Science Center, Memphis, TN, United States; ^2^Evolution and Genomic Sciences, Faculty of Biology, Medicine, and Health, Manchester Academic Health Science Centre, The University of Manchester, Manchester, United Kingdom

**Keywords:** schizophrenia, prepulse inhibition, *APBB1IP*, cross-species, comparative analysis, BXD, endophenotype

## Abstract

**Background**: Prepulse inhibition (PPI) of the startle response is a highly conserved form of sensorimotor gating, disruption of which is found in schizophrenia patients and their unaffected first-degree relatives. PPI can be measured in many species, and shows considerable phenotypic variation between and within rodent models. This makes PPI a useful endophenotype. Genome-wide association studies (GWAS) have been carried out to identify genetic variants underlying schizophrenia, and these suggest that schizophrenia is highly polygenic. GWAS have been unable to account for the high heritability of schizophrenia seen in family studies, partly because of the low power of GWAS due to multiple comparisons. By contrast, complementary mouse model linkage studies often have high statistical power to detect variants for behavioral traits but lower resolution, producing loci that include tens or hundreds of genes. To capitalize on the advantages of both GWAS and genetic mouse models, our study uses a cross-species approach to identify novel genes associated with PPI regulation, which thus may contribute to the PPI deficits seen in schizophrenia.

**Results**: Using experimental data from the recombinant inbred (RI) mouse panel BXD, we identified two significant loci affecting PPI. These genomic regions contain genetic variants which influence PPI in mice and are therefore candidates that may be influencing aspects of schizophrenia in humans. We next investigated these regions in whole-genome data from the Psychiatric Genomics Consortium (PGC) schizophrenia GWAS and identify one novel candidate gene (*ABPP1IP*) that was significantly associated with PPI in mice and risk of schizophrenia in humans. A systems genetics approach demonstrates that *APBB1IP* coexpresses with several other genes related to schizophrenia in several brain regions. Gene coexpression and enrichment analysis shows clear links between *APBB1IP* and the immune system.

**Conclusion**: The combination of human GWAS and mouse quantitative trait loci (QTL) from some of the largest study systems available has enabled us to identify a novel gene, *APBB1IP*, which influences schizophrenia in humans and PPI in mice.

## Background

Schizophrenia is a severe mental disorder characterized by hallucinations, delusions, social withdrawal, and cognitive deficits such as poor executive function and working memory (Burmeister et al., 2008). It is a major cause of morbidity contributing significantly to the global burden of disease (Lopez et al., 2006) and affects about 1% of the population. While onset typically occurs after the period of brain development that follows puberty in late adolescence or early adult life (Kirkbride et al., 2012), there is now strong clinical and epidemiological evidence that schizophrenia reflects a disturbance of neurodevelopment (Owen et al., 2016). Both genetic and environmental risk factors for schizophrenia have been identified that are linked to immune function, e.g., (Murray et al., 2019), an idea that was first postulated more than a century ago (for a review see Khandaker et al., 2015). The brain is no longer considered an immunologically privileged site, isolated from the immune system *via* the blood-brain barrier (Carson et al., 2006). Microglia derived from the hemopoietic system beyond the central nervous system (CNS), make up about 10% of the brain cell mass, equal to that of neurons (Hanisch and Kettenmann, 2007). Animal models provide evidence that systemic inflammatory cytokines, e.g., interleukin 6 (IL6) or tumor necrosis factor-α (TNF-α), communicate with the brain, affecting synaptic plasticity, cortisol concentrations, neurotransmitters, activation of the hypothalamic-pituitary-adrenal axis, and increased oxidative stress in the brain (Dantzer et al., 2008; Khandaker et al., 2015; Murray et al., 2019). These effects could contribute to changes in mood, cognition and behavior, supported by epidemiological studies that suggest links between schizophrenia and systemic inflammation (Brown and Derkits, 2009; Miller et al., 2011; Khandaker et al., 2014).

In order to understand the etiology and biology of schizophrenia it is essential to identify the underlying causal variants. This will allow us to link the disorder to specific proteins and pathways, opening the door for therapeutic targets, and a more accurate prediction of genetic predisposition.

Twin studies estimate broad heritability of schizophrenia at ~81% (Sullivan et al., 2003; Craddock et al., 2005), and heritability of 31–44% is reported for nuclear and extended families (Light et al., 2014) indicating a substantial genetic component; however the role of genetic variation in the etiology of schizophrenia is complex. A recent, large-scale genome-wide association study (GWAS) identified 108 independent genomic loci exhibiting genome-wide significance associated with schizophrenia, suggesting that schizophrenia is highly polygenic, with many single nucleotide polymorphisms (SNPs), each of small effect (Schizophrenia Working Group of the Psychiatric Genomics Consortium, 2014). But GWAS have been unable to account for the large portion of the heritability shown by twin and family studies. To reduce type 1 error and account for association testing of ~1 million SNPs in the human genome, multiple testing corrected significance threshold of *p*-value ≤5 × 10^−8^ is required (Barsh et al., 2012). This stringent significance threshold leads to many false-negative associations, potentially preventing the discovery of additional loci that could potentially offer important insights. Despite the modest statistical power due to high multiple testing correction, loci in human GWAS are defined with high precision, potentially to the individual SNP level. In contrast, mouse model linkage studies are often statistically powered enough to detect genetic effects but have lower resolution, producing loci that include tens or hundreds of genes (e.g., Ackert-Bicknell et al., 2010; Hager et al., 2012). Mouse systems have been extensively used to as a gateway to experimental precision medicine aiming to establish the genetic basis of disease traits due to the high degree of homology between mouse and human genomes, and because of the possibility of precisely manipulating the mouse genome and experimental variables (Peters et al., 2007). For example, recombinant inbred (RI) mouse models, such as the BXD family used here, have the advantage that the same genome can be replicated an unlimited number of times, allowing both invasive tissue collection, and for tissue to be collected along a developmental timeline. To capitalize on the advantages of both GWAS and genetic mouse models we and others have developed methods that combine data from both systems, gaining power from mouse model studies and precision from human GWAS (Himes et al., 2013; Ashbrook et al., 2014, 2015; Jing et al., 2016; Mignogna et al., 2018).

Prepulse inhibition (PPI) of the startle response is a form of sensorimotor gating that occurs when a weak, subthreshold “prestimulus” presented 30–500 ms prior to an intense startling stimulus inhibits the startle response (Swerdlow et al., 2000). PPI is a highly conserved phenomenon and observed across sensory systems, but acoustic PPI is frequently used for investigations and is usually measured from the eyeblink response (Swerdlow et al., 1999). PPI deficits were first observed in schizophrenia patients over 40 years ago (for a review see Braff et al., 2001), but are also evident in their unaffected first degree relatives (Cadenhead et al., 2000), and patients with schizotypal personality disorder (Cadenhead et al., 1993), reflecting a genetically transmitted susceptibility to sensorimotor gating deficits. In fact, PPI heritability has been estimated at 32% (Greenwood et al., 2007), similar to the schizophrenia heritability estimates of 31% and 44% for nuclear and extended families. This suggests similar heritabilities for both the disease and the endophenotype (Light et al., 2014). In terms of genetic analysis, endophenotypes are heritable, measurable biomarkers that are potentially less genetically complex (Gottesman and Gould, 2003). PPI deficits are not unique to schizophrenia however, and have been observed in a number of psychiatric and neurological disorders including bipolar disorder, autism, obsessive-compulsive disorder, Tourette’s syndrome and Huntington’s disease (Kohl et al., 2013), but are most widely replicated in schizophrenia patients (Braff et al., 2001). Thus PPI meets the criteria for a viable endophenotype, outlined by Turetsky et al. (2007), which specifies that the endophenotype must be associated with a disorder, heritable, evident in unaffected family members, easily measured, with high test, re-test reliability. This, combined with the observation that inbred mouse strains demonstrate considerable phenotypic variation in PPI (for a review see Petryshen et al., 2005), renders PPI a behavioral phenotype with rich potential for discovery of genes in genetic mouse models that confer risk or resistance to schizophrenia.

Animal models have proven invaluable in genetics research and previous research has demonstrated the effectiveness of using a cross-species approach to identify genes underlying specific traits (Himes et al., 2013; Ashbrook et al., 2014, 2015; Jing et al., 2016; Mignogna et al., 2018). The benefit of this approach is that it allows the investigation of phenotypes without requiring the quantification of experimental perturbations on the biological system. Our study uses a cross-species approach to identify genes associated with PPI regulation, which thus may contribute to the PPI deficits seen in schizophrenia. We use data obtained from populations that have undergone segregation analysis for large numbers of common sequence variants and associated differences in phenotypes (Ashbrook et al., 2014, 2015). First, we used data from PPI in the most deeply phenotyped mammalian model system, the RI mouse panel BXD (Wu et al., 2014), to identify quantitative trait loci (QTL). This identifies areas of the genome containing genetic variants that influence PPI in mice and therefore may be influencing aspects of schizophrenia in humans. We then went on to investigate these genomic regions in a large human GWAS of schizophrenia, the SNP summaries of which are available in an online database (Schizophrenia Working Group of the Psychiatric Genomics Consortium, 2014). We identified one gene (amyloid-β precursor protein-binding family B member 1 interacting protein; *APBB1IP)* that was significantly associated with PPI in mice and the risk of schizophrenia in humans at the genome-wide level. Our systems genetics approach demonstrates that *APBB1IP* coexpresses with several other genes related to schizophrenia in several brain regions, providing a potential mechanism of action.

## Materials and Methods

### Mouse and Human Data

The BXD RI panel was derived by crossing the parental B6 and D2 strains followed by >20 generations of inbreeding, and currently consists of ~150 commercially available and densely genotyped strains (Williams et al., 2001; Peirce et al., 2004; Ashbrook et al., 2019). The BXDs are the most deeply phenotyped mammalian experimental population, and have been used as a genetic reference panel for over 40 years (Morse et al., 1979). The online resource www.genenetwork.org contains BXD genotypes and software for rapid QTL mapping (GeneNetwork). This resource also contains over 7000 BXD phenotypes uploaded by researchers that can be compared and assessed for genetic correlations and common QTL. These phenotypes include strain mRNA expression levels for many tissues that can be utilized for systems genetics analysis and prioritization of positional candidate genes in behavioral and physiological QTL (Bubier and Chesler, 2012). For our analysis, we used data on PPI startle (Bagley, 2017), available in GeneNetwork (Sloan et al., 2016). We selected any trait with at least one significant QTL (*p* ≤ 0.05) at the genome-wide level. Significance was calculated within GeneNetwork, using 5,000 permutations of trait values and genotypes. A *p*-value of 0.05 is defined as a likelihood ratio statistic (LRS) score greater than 95% of the permutated datasets (Wang et al., 2003).

Human GWAS data for schizophrenia were obtained from the Psychiatric Genomics Consortium (PGC; Schizophrenia Working Group of the Psychiatric Genomics Consortium, 2014), containing 36,989 cases and 113,075 controls. The Knowledge-Based Mining System for Genome-wide Genetic Studies (KGG^1^; version 4.0) was used to convert the SNP *p*-values to gene *p*-values using the GATES method, an extension of a Simes test that integrates functional information and association evidence to rapidly combine the *p*-values of the SNPs within a gene, to produce valid gene-based *p*-values (Li et al., 2011). The GATES test is advantageous because it requires only summary GWAS and ancestral population linkage disequilibrium (LD) data; it provides effective control of the type 1 error rate regardless of gene size and LD pattern among markers, and permutation or simulation is not required to evaluate empirical significance (Li et al., 2011). Gene locations were from the Hg19 genome build. We used the complete set of data from the PGC GWAS for schizophrenia (PGC-GWAS; ckqny.scz2snpres.gz), and ancestry matched genotypes from phase 3 of 1,000 Genomes Project to adjust LD between variants (Li et al., 2014; 1 kg.phase3.v5.shapeit2.eur.hg19.tar). For each gene in Hg19 a GATES *p*-value was calculated for association with schizophrenia.

For the joint mouse-human analysis, the human homologs of genes within the mouse QTL were identified using Ensembl version 94 (Zerbino et al., 2017). To assess if any particular gene is associated with both PPI in mouse and schizophrenia in humans, we examined the GATES derived *p*-values of human homologs of genes within the significant BXD QTL. The human GWAS significance values were Bonferroni corrected for multiple comparisons using the total number of homologous genes (Ashbrook et al., 2014, 2015).

### Gene Ontology

Genease (Genease) and WebGestalt (Zhang et al., 2005) tools were used for Gene Ontology (GO) annotation. For microarray-based transcriptome data used in enrichment analyses in WebGestalt, the array IDs were used, and the total probes on the array were used as background. For RNA-seq data, ENTREZ IDs were used, and all protein-coding genes were used as background.

## Results

We began by identifying QTL for PPI in the BXD RI family. Next, we investigated the homologous genomic regions in human schizophrenia GWAS data and were able to identify one novel candidate gene, *APBB1IP* (also referred to as *PREL1, RARP1*, and *RIAM*; Entrez 54518).

### QTL for Schizophrenia-Related Phenotypes in Mice

**Figure 1:**
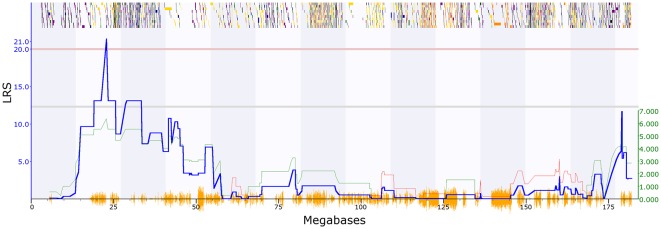
Quantitative trait loci (QTL) map showing significant QTL on chromosome 2 between 22 and 24 Mbp, prepulse inhibition (PPI) trait (GeneNetwork ID 18755). The red horizontal line towards the top of the figures illustrates the genome-wide significance threshold, which is *p*-value ≤ 0.05 genome-wide corrected [significant likelihood ratio statistic (LRS) = 20.54]. The lower, blue line indicates the significance of the trait at each position along the genomic regions. The lower, green and red line shows the negative additive coefficient. Regions where DBA/2J alleles increase trait values are shown in green, and regions where the C57BL/6J alleles increase trait values are shown in red, with the scale in green on the left. The multi-colored blocks at the top of the figure represent genes, showing where they fall in the genomic region, and their length. The position of segregating single nucleotide polymorphisms (SNPs) in the BXD family are shown by the orange Seismograph track at the bottom of each map. Adapted from genenetwork.org.

**Figure 2:**
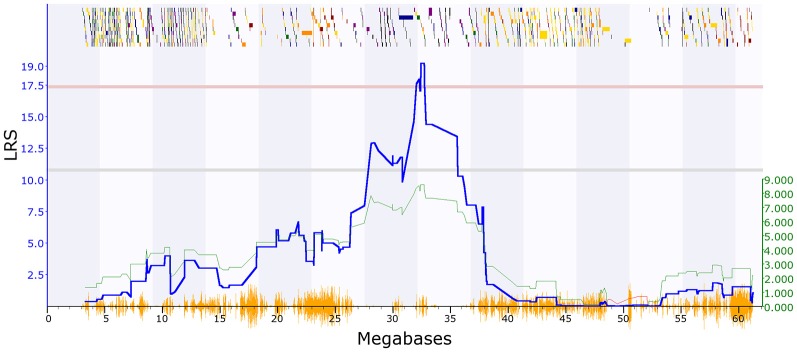
QTL map showing a significant QTL on chromosome 19 between 31 and 33 Mbp, PPI trait (GeneNetwork ID 11428). The red horizontal line towards the top of the figures illustrates the genome-wide significance threshold, which is *p*-value ≤ 0.05 genome-wide corrected (significant LRS = 17.30). The lower, blue line indicates the significance of the trait at each position along the genomic regions. The lower, green and red line shows the negative additive coefficient. Regions where DBA/2J alleles increase trait values are shown in green, and regions where the C57BL/6J alleles increase trait values are shown in red, with the scale in green on the left. The multi-colored blocks at the top of the figure represent genes, showing where they fall in the genomic region, and their length. The position of segregating SNPs in the BXD family are shown by the orange seismograph track at the bottom of each map. Adapted from genenetwork.org.

PPI deficits are associated with schizophrenia (Braff et al., 2001), and we, therefore, investigated genetic variation in this trait. Two PPI traits met our criteria in GeneNetwork: GN18755 (trait description: Acoustic startle response, PPI, change in startle response with 74 dB prepulse from response with no prepulse in females, unstressed controls), which has one significant QTL with a maximum LRS of 21.4 on chromosome 2 (Figure 1). The second trait (GN11428: Acoustic startle response, PPI at 85 dB for males; Philip et al., 2010), has one significant QTL on chromosome 19 with a maximum LRS of 19.2 (Figure 2).

To further characterize the two QTL we used Genome-wide Efficient Mixed Model Association (GEMMA) software (Zhou and Stephens, 2012) and Leave One Chromosome Out (LOCO) in GeneNetwork2^2^. For GN18755 the most significant marker was rs13476379 at chromosome 2: 22.82 Mb. This marker has a −log(*p*) value of 6.16, compared to the surrounding marker values of 4.13. This marker lies within the *Apbb1ip* gene. For GN11428, the most significant marker was rs6197068 at chr19: 32.74 Mb. This marker has a −log(*p*) value of 5.69. This lies between the genes *Atad1* and *Pten*.

The QTL on proximal chromosome 2 (22.62–22.86 Mbp) contains four genes with human homologs, and the QTL on proximal chromosome 19 (31.95–32.75 Mbp) contains eight genes with human homologs, all of which are on chromosome 10 (Table 1). We then examined the PGC Schizophrenia GWAS data to identify variants within the homologous region significantly associated with schizophrenia. Only one of the twelve genes, *APBB1IP*, has a nominally significant *p*-value (*p* = 0.0117).

**Table 1:** Human homologs (genome assembly: GRCh38.p12) of the 12 mouse genes (Genome assembly: GRCm38.p6) contained in the genome-wide significant quantitative trait loci (QTL) of prepulse inhibition (PPI) phenotypic traits 18755 and 11428.

Trait ID	Gene stable ID—Mouse	Gene name	Position	Gene stable ID—Human Homolog	Gene name	Position
18755	ENSMUSG00000058835	Abi1	Chr2:22.94-23, 04	ENSG00000136754	ABI1	Chr10:26.75-26.86
18755	ENSMUSG00000026786	Apbb1ip	Chr2:22.77-22.88	ENSG00000077420	APBB1IP	Chr10:26.44-26.57
18755	ENSMUSG00000026787	Gad2	Chr2:22.62-22.69	ENSG00000136750	GAD2	Chr10:26.22-26.30
18755	ENSMUSG00000026784	Pdss1	Chr2:22.90-22.94	ENSG00000148459	PDSS1	Chr10:26.70-26.75
11428	ENSMUSG00000052595	A1cf	Chr19:31.87-31.95	ENSG00000148584	A1CF	Chr10:50.80-50.89
11428	ENSMUSG00000013663	Pten	Chr19:32.76-32.83	ENSG00000171862	PTEN	Chr10:87.86-87.97
11428	ENSMUSG00000024887	Asah2	Chr19:31.98-32.06	ENSG00000188611	ASAH2	Chr10:50.18-50.25
11428	ENSMUSG00000013662	Atad1	Chr19:32.67-32.71	ENSG00000138138	ATAD1	Chr10:87.75-87.84
11428	ENSMUSG00000024896	Minpp1	Chr19:32.49-32.52	ENSG00000107789	MINPP1	Chr10:87.50-87.55
11428	ENSMUSG00000024899	Papss2	Chr19:32.62-32.67	ENSG00000198682	PAPSS2	Chr10:87.66-87.75
11428	ENSMUSG00000024887	Asah2	Chr19:31.98-32.06	ENSG00000204147	ASAH2B	Chr10:50.74-50.82
11428	ENSMUSG00000040451	Sgms1	Chr19:32.12-32.39	ENSG00000198964	SGMS1	Chr10:50.31-50.63
11428	ENSMUSG00000052595	A1cf	Chr19:31.87-31.95	ENSG00000148584	A1CF	Chr10:50.80-50.89
11428	ENSMUSG00000013663	Pten	Chr19:32.76-32.83	ENSG00000171862	PTEN	Chr10:87.86-87.97

Next, we checked for associations in an independent dataset, the UKBiobank collection using PheWeb (PheWeb). This shows that two variants in *APBB1IP* are significantly associated with self-reported schizophrenia (UKBB ID 20002_1289). The first is rs570949810 at chr10: 26,823,566 (T/G, *p* = 1.8e-9) and the second is rs554788824 at chr10:26,818,850 (G/A, *p* = 8e-9). Both variants are also associated with usage of two antipsychotics used to treat schizophrenia, trifluoperazine (*p* = 1.1e-6 and 5.7e-7, respectively) and amisulpride (*p* = 7.2e-7 and 1.3e-5, respectively). This further supports *APBB1IP* as a schizophrenia associated gene.

These results thus provide strong evidence that *APBB1IP* influences schizophrenia-related traits in both mouse and human. We can next take advantage of massive gene expression datasets to identify plausible mechanisms by which* APBB1IP* may be linked to schizophrenia.

### Human Gene Expression

**Figure 3:**
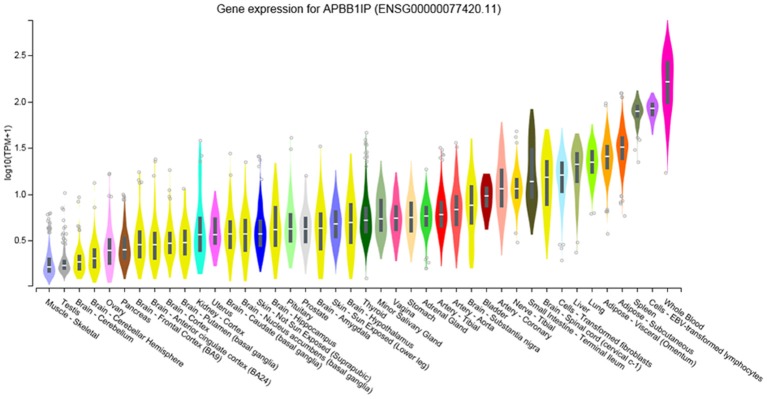
Gene Expression of APBB1IP in Human Tissues taken from www.gtexportal.org (Lonsdale et al., 2013). Expression values are shown in log-transformed Transcripts Per Million (TPM). These TPM values were calculated from a gene model with isoforms collapsed to a single gene. We did not apply any other normalization steps. Violin plots are shown as median and 25th and 75th percentiles. Outliers above or below 1.5 times the interquartile range are displayed as points. Adapted from www.gtexportal.org.

To identify the regions in which our candidate gene is expressed we used the Genotype-Tissue Expression (GTEx) portal^3^. The GTEx portal indicates that *APBB1IP* has high expression in whole blood, in lymphocytes and in the spleen (Figure 3). This links *APBB1IP* to the immune system, confirming previous findings (Zhang and Wang, 2012; Su et al., 2015; Patsoukis et al., 2017). To expand on this, we used GeneNetwork to query the GTEXv5 Human Whole Blood (GN770) data and searched for genes that are significantly coexpressed with *APBB1IP* in whole blood. There are 3464 genes which show coexpression (*r* ≥ 0.5 or ≤ −0.5, *p* ≤ 0.05; Supplementary Table S1), and enrichment analysis of these genes shows clear links to the immune system (e.g., regulation of immune response, GO:0050776, FDR = 5.19e-10; Innate Immune System, R-HSA-168249, FDR < 1e-20; Supplementary Figure S1, Supplementary Table S2). Interestingly, the top two enriched drug targets interact with ARP2/3, which has previously been linked to schizophrenia (Gokhale et al., 2016; Yan et al., 2016; FDR = 7.96e-05 for both; Supplementary Table S3).

However, GTEx only uses adult tissue (20–71 years old; Lonsdale et al., 2013), and schizophrenia is known to involve neurodevelopmental deficits, which would occur before this age range. As such, we examined the Allen Brain Atlas Human Brain (Hawrylycz et al., 2012) and Developing Human Brain (Miller et al., 2014) data. The Developing Brain data suggests that *APBB1IP* expression increases postnatally in a number of brain areas (Supplementary Table S4) and that in the adult brain there is higher expression in the cerebellum and dentate gyrus, compared with other brain areas. Both of these structures have been previously linked to schizophrenia (Yuan et al., 2015; Mothersill et al., 2016; Nakao et al., 2017). Given these links, we then repeated the above analysis, using the cerebellar and hippocampal GTEXv5 data in GeneNetwork. In the cerebellum, 282 genes coexpress with *APBB1IP* (*r* ≥ 0.5 or ≤ −0.5, *p* < 0.05; Supplementary Table S5). Again, we see enrichment for immune-related genes (e.g., regulation of immune system process, GO:0002682, FDR = 1.9e-12; TYROBP Causal Network, WP3945, FDR < 1e-20; Supplementary Figure S2; Supplementary Table S6). This is of further interest as TYROBP has also been linked to schizophrenia (de Baumont et al., 2015).

Finally, we used GeneFriends to examine the potential roles of *APBB1IP*, which is agnostic to tissue type. Here, we found that 785 genes commonly coexpress with *APBB1IP* (*r* ≥ 0.5 or ≤ −0.5; Supplementary Table S7). The enrichment analysis suggests strong involvement with immune-related processes (Supplementary Figure S3).

We have thus strong evidence that, in humans, *APBB1IP* is linked to immune function, and is expressed in the developing brain. In the next step, we use murine gene expression data to explore the cross-species translatability of this finding, which may enable future work to develop interventions that can be tested in mouse models.

### Mouse Gene Expression

**Figure 4:**
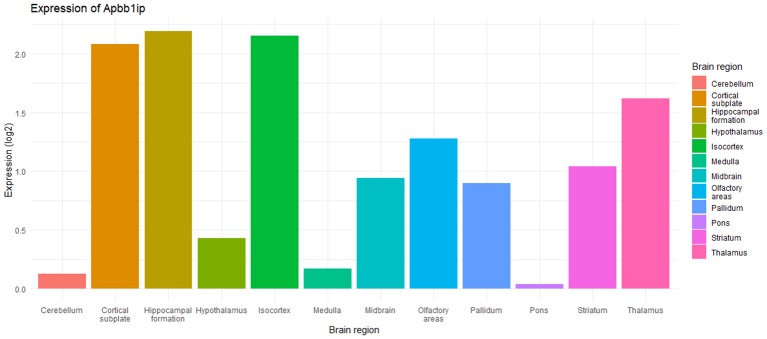
Bar graph showing expression of *Apbb1ip* across different brain regions in young adult mice, adapted from Allen Brain Atlas (Brain-Map; Lein et al., 2007; Sunkin et al., 2013; Zaldivar and Krichmar, 2014). The data is from *Apbb1ip* probe RP_050111_02_C07, with data taken from a sagittal cut from a 56-day old male C57BL/6J.

**Figure 5:**
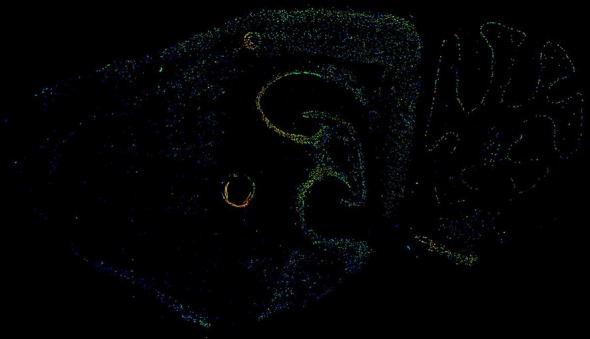
Image of expression of *Apbb1ip* in the young adult mouse brain, taken from the Allen Brain Atlas (Brain-Map; Lein et al., 2007; Sunkin et al., 2013; Zaldivar and Krichmar, 2014). The data is from *Apbb1ip* probe RP_050111_02_C07, with data taken from a sagittal cut from a 56-day old male C57BL/6J.

We first repeated the same GeneFriends analysis as above but using mouse data. This showed 179 commonly coexpressed genes (*r* ≥ 0.5 or ≤ −0.5; Supplementary Table S8), showing that genes that commonly coexpress with *Apbb1ip* are involved in immune response (Supplementary Figure S4). Next, we sought to establish if *Apbb1ip* is expressed in homologous tissues to *APBB1IP*. The Allen Brain Atlas shows expression of *Apbb1ip* across a number of brain regions in young adult mice (Figures 4, 5), analogous to the human brain. In particular, we again see the expression in the dendrite gyrus, as we saw in humans. Changes in gene expression in the dendrite gyrus in schizophrenia have been reported by others (Tavitian et al., 2019).

In the next step of our analysis, we analyzed deep gene expression data available for the BXD family. This enables us to map expression QTL (eQTL) to identify regions of the genome that are regulated by our gene of interest. Second, we can examine gene co-expression to find groups of genes which are under similar regulatory control and are therefore likely to be involved in the same biological processes. Thirdly, we can detect low levels of gene expression, or gene expression that only occurs under specific conditions.

Because of the high expression of *APBB1IP* in human lymphocytes, we first used two lymphocyte cell populations from the BXD family, T-helper cells and T-regulatory cells. In both cell types there is a *cis*-eQTL, indicating that a variant in *Apbb1ip* regulates expression of this gene, although this only reaches genome-wide significance in the T-helper cells (LOD = 7.8, 1.5 LOD confidence interval chromosome 2:17.95–23.77 Mb), and reaches chromosome-wide significance in T-regulatory cells. We next looked for the probes that correlated significantly with the expression of *Apbb1ip* (*p* < 0.05) in both of these datasets, to find a co-regulated network. In T-helper cells, 3,623 probes (3,249 genes; Supplementary Table S9) were co-expressed with *Apbb1ip* and in T-regulatory cells 2,565 (2,367 genes; Supplementary Table S10). Of these, 507 genes were co-expressed in both cell types, and gene-set enrichment was carried out on these 507 using WebGestalt. The most highly enriched annotations were cell migration (GO:0016477, FDR = 6.73e-03) and cell motility (GO:0048870, FDR = 6.73e-03). Interestingly, cell migration has been found to be dysregulated in schizophrenia patient-derived cells (Tee et al., 2017).

We next moved on to investigate more broadly all tissues with transcriptome data in the BXD. For each dataset, we searched for genome-wide significant eQTL, and recorded their position. To reduce false positives, we concentrated only on that eQTL which occurred in more than one dataset. We found the same *cis*-eQTL location that we detected in T-helper cells in a number of tissues (pituitary, aged hippocampus, midbrain, spleen, gastrointestinal tract, hypothalamus), showing that a variant near *Apbb1ip* influences its expression across tissues. This does not mean that this variant is not expressed in tissues other than those given above, but simply that no effect could be detected in that particular dataset. For each of these issues with a confirmed* Apbb1ip cis*-eQTL, we carried out a coexpression analysis. If the tissue contained multiple probes for *Apbb1ip* then these were combined together using a PCA to produce an eigengene (Ringnér, 2008), which was then correlated against the whole dataset for that tissue.

We found that the midbrain (GN381) had a very strong cis-eQTL (chromosome 2, 17.95–23.77 Mb, peak LOD = 11.75). As this clearly showed that *Apbb1ip* controls its own expression, we then looked for genes that coexpress with *Apbb1ip*. There were 3,093 probes which correlated with *Apbb1ip* expression, representing 1,607 unambiguously mapped genes (Supplementary Table S11). These genes showed significant enrichment for immune annotations, including immune response (GO:0006955, FDR = 3.25e-10; Supplementary Figure S5). This shows that the immune gene coexpression seen in the human brain is also seen in the murine brain.

Further, in the gastrointestinal data (Supplementary Table S12), there was significant enrichment for a range of immune-related annotations (e.g., immune system process, GO:0002376, FDR = 0e+00; Supplementary Figure S6). When correlated with the phenotypes database on GN, the most significant correlation was with visual discrimination learning using a touchscreen assay, mean reaction time to nose-poke in 3-month-old males (GN 16210, rho = −0.8137, *p* = 2.06e-05, *N* = 17). This is of interest, as reaction time is known to be disrupted in schizophrenia patients and their relatives (Chrobak et al., 2017).

In the hypothalamus data (GN159) we saw a significant *cis*-eQTL (19.02–36.10 Mb). There was significant coexpression with 17,960 probes, mapping to 9,160 genes, and once again we found immune system processes in the enrichment analysis (GO:0002376, FDR < 1e-20; Supplementary Figure S7). We also identified significant enrichment for focal adhesion-related annotations (mmu04510, FDR = 1.15e-08; WP85, FDR = 3.07e-07; Supplementary Table S13), previously shown to be altered in schizophrenia (Fan et al., 2013). The highest correlating phenotype was an infectious disease, immune system phenotype (rho = 0.7326, *p* = 4.71e-06, *n* = 27; GN 15561).

Pituitary (GN335) had nine probes with strong cis-eQTLs, so these were combined into an eigengene, and remapped to produce a cis-eQTL with a max LOD of 14.55 running from 19.02 to 23.77Mb. Once again, amongst the coexpressed genes (Supplementary Table S14) we saw enrichment for the immune system process (GO:0002376, FDR < 1e-20; Supplementary Figure S8). We also saw focal adhesion as significant again (mmu04510, FDR = 7.37e-07; Supplementary Table S15).

In the spleen (GN227), 3,700 probes, mappable to 2,846 genes, were significantly correlated with *Apbb1ip* (Supplementary Table S16). Unlike the majority of other tissues, and more similar to the T-cell data, the most enriched annotations were associated with cell cycle (GO:0007049, FDR = 1.5e-04; Supplementary Figure S9).

Given previous research suggesting striatal expression of genes affecting mental disorders (Ashbrook et al., 2015), and strong evidence for shared genetics between mental disorders (Cross-Disorder Group of the Psychiatric Genomics Consortium, 2013), we next analyzed striatal expression of *Apbb1ip* using a transcriptome dataset for postnatal day 14 mice. Again, we used GeneNetwork for QTL mapping and identified three probes for *Apbb1ip*, which show a strong QTL on chromosome 2, between 88 and 98 Mbps. We computed the 1st principal component of these three probe sets (an eigengene, Ringnér, 2008), and mapped this. This produced a peak LOD of 5.58 at 97.32 Mb, with a confidence interval from 87.46 to 99.75 Mbp. This region contains 146 genes, 88 of which contain SNPs or indels. We then narrowed down these genes to those which showed a significant *cis*-eQTL (indicating they regulate their own expression), and the expression of which correlated with *Apbb1ip*. These genes were *Morf4l1, Mthfs, 1600029I14Rik, Plscr4, 1190002N15Rik, Armc8, Zbtb38, Faim, Rbp1, C730029A08Rik, Zic1, Trpc1, Adamts7*, and *2810026P18Rik*. Of these, *Plscr4, Armc8, Zbtb38, Rbp1, Zic1*, and *Trpc1* have been linked to schizophrenia or schizophrenia-related disorders in previous studies (Kim et al., 2007; Li et al., 2017). For example, *Zic* genes are strongly expressed in the cerebellum and have been shown to mediate cerebellar development (Aruga and Millen, 2018), and behavioral abnormalities of *Zic1* mutant mice can serve as models for diseases involving sensorimotor gating abnormalities, such as schizophrenia (Ogura et al., 2001).

We also discovered a strong eQTL in erythroid cells (GN150), chromosome 2:81.02–93.32, i.e., overlapping with the eQTL which we observed in the striatum. There are 5,830 probes (Supplementary Table S17), representing 4472 unambiguously mapped genes, and these genes were enriched for annotations such as RNA processing (GO:0006396, FDR < 1e-20) and translation (GO:0006412, FDR < 1e-20).

### Enrichment of Schizophrenia-Associated Genes

Although we have identified genes that are coexpressed with *Apbb1ip* and are enriched for annotations linked to schizophrenia, this does not directly link them with schizophrenia. We thus performed a permutation analysis to look specifically for enrichment for genes that have been previously associated with schizophrenia.

We used the GWAS catalog for schizophrenia (GWAS-Catalog) to identify known associations. This had information for 2008 associations, however for some of these no genes were reported (*n* = 708) and for many, several potential genes were reported (i.e., genes within the LD block of the associated SNP). These associations represented 1326 unique human genes, of which 959 had murine homologs. For each of the above tissues, we produced a full list of genes examined in the entire dataset (e.g., all genes on a microarray or that were recorded in an RNA-seq experiment). For each tissue, we recorded the overlap in genes coexpressed with *Apbb1ip* and with genes associated with schizophrenia. We then performed 100,000 permutations, taking random subsets of the same length as the coexpressed genes, and recording their overlap with the schizophrenia genes. The number of times that the random genes had a higher overlap than the co-expressed genes, divided by 100,000, produced an empirical *p*-value.

Table 2: enrichment for schizophrenia-associated genes in *Apbb1ip* coexpression genes from mouse and human tissue. The species and tissue used for coexpression are shown, the significance of the overlap between genes associated with schizophrenia and genes coexpressed with *Apbb1ip*, the number of genes which overlap between genes associated with schizophrenia and genes coexpressed with *Apbb1ip*, the total number of genes which coexpressed with *Apbb1ip* and the total number of schizophrenia-associated genes included in the analysis (i.e., the number of schizophrenia-associated genes which were included in the expression dataset).

**Table 2:** Permutation analysis of the overlap between genes coexpressing with *Apbb1ip* in mouse tissues, and genes associated with schizophrenia in genome-wide association studies (GWAS).

Species	Tissue	Significance of overlap (*p*-value)	Number of overlapping genes	Number of coexpressing genes	Number of schizophrenia genes included in analysis
mouse	Spleen	<1e+06	129	829	250
mouse	T-regulator cells	<1e-06	108	1,539	571
mouse	Striatum PND14	1.80E-05	336	6,932	913
mouse	Hypothalamus	3.00E-05	432	8,680	928
mouse	Pituitary	8.03E-03	361	7,666	928
mouse	T-helper cells	0.105	139	2,945	833
mouse	Aged hippocampus	0.212	116	2,597	916
mouse	Midbrain	0.432	72	1,840	928
mouse	GI tract	0.915	113	2,929	896
human	Whole blood	<1e+06	137	3,464	1,075
human	Nucleus accumbens	<1e+06	209	4,430	1,075
human	Spleen	3.70E-05	27	598	1,075
human	Cerebellum	0.000137	16	282	1,075
human	Hippocampus	0.237	4	130	1,075

*The tissue, the significance of overlap, the number of overlapping genes, the total number of genes that coexpressed, and the number of schizophrenia genes included in the analysis*.

## Discussion

We have identified *APBB1IP*, on chromosome 10 in humans and chromosome 2 in mice, as a novel gene that influences schizophrenia in humans and PPI in mice, and identified a number of genes which may be involved in this association. *APBB1IP* is found to have high expression in the whole blood, lymphocytes and the spleen, linking it to the immune system. Coexpression and enrichment analyses in the whole blood, cerebella and hippocampus showed enrichment for immune-related genes. Similar enrichment in immune-related processes showed that genes commonly coexpressing with *Apbb1ip* are also involved in immune responses in the mouse.

Our results suggest that *APBB1IP* is involved in cell motility, the immune response and neuronal development, all of which have been linked to schizophrenia. *Apbb1ip* is found to be expressed in homologous tissues to *ABPP1IP*, particularly the dentate gyrus, previously linked to schizophrenia (Yuan et al., 2015; Nakao et al., 2017). Immune gene coexpression is seen in the human brain and the murine brain. This confirms the translatability of this gene expression and supports mouse models as useful tools in which interventions can be tested. Several brain regions of potential interest, including the hippocampus and cerebellum, did not show any eQTL for *Apbb1ip* in the BXD population. This could have several explanations. First, it could be that *Apbb1ip* is under the regulation of many loci of small effect, which cannot individually be detected with the number of strains used. Another possibility is that the strains used for the particular gene expression study did not segregate for a variant which alters *Apbb1ip* expression. Finally, it could be that the timing was wrong and that *Apbb1ip* was not being differentially expressed at the time point expression was measured.

The potential mechanism for the link between *ABPP1IP* and an immune response is more speculative. Evidence suggests that T cells may play an important role in the pathophysiology of schizophrenia. Acute psychosis has been associated with the increased frequency of activated lymphocytes within the cerebrospinal fluid compared with controls (Nikkilä et al., 2001). ABPP1IP has been identified as a Rap1-binding protein important for leukocyte integrin activation (Lafuente et al., 2004). *Rap1* has previously been identified as interacting with candidate schizophrenia susceptibility gene, *Rapgef6*, disrupting amygdala function in mice (Levy et al., 2015). Inflammation triggers leukocyte recruitment into site-specific tissue. Leukocyte integrins play a critical role in leukocyte recruitment and have thus emerged as key molecules in potential therapeutic targets for immune-mediated or inflammatory diseases (Yonekawa and Harlan, 2005). Regulation of lymphocyte adhesion to endothelium and trafficking of lymphocytes to secondary lymphoid organs is critical for adaptive immunity (Mor et al., 2007). Studies in ABPP1IP mice show that they are viable, fertile and apparently healthy (Stritt et al., 2015), but peripheral lymph nodes were depleted of both B and T cells resulting in abnormal lymphocyte trafficking and defective humoral immunity to T-cell-dependent antigens (Su et al., 2015). While systematic investigations of the humoral immune system in schizophrenia are lacking, there is some research suggesting that this “unspecific” arm of the immune system may be more activated in schizophrenic patients than in controls (Wilke et al., 1996; Müller et al., 1998). Activation of this system can be found mainly in unmedicated schizophrenic patients, which suggests it may reflect the disease process rather than the antipsychotic treatment.

## Conclusion

In summary, the combination of human GWAS and mouse QTL from some of the largest study systems available has enabled us to identify a novel gene, *APBB1IP*, which influences schizophrenia in humans and PPI in mice.

## Data Availability Statement

The datasets analyzed during the current study are available in the GeneNetwork repository, http://www.genenetwork.org/webqtl/main.py; the NHGRI-EBI Catalog of published Genome-wide association studies (GWAS), https://www.ebi.ac.uk/gwas/efotraits/EFO_0000692. The GTEx Project was supported by the Common Fund of the Office of the Director of the National Institutes of Health, and by NCI, NHGRI, NHLBI, NIDA, NIMH, and NINDS. The data used for the analyses described in this manuscript were obtained from www.gtexportal.org, the GTEx Portal on 04/06/2019.

## Author Contributions

RH, SC and DA conceptualized and designed the study. SC and DA carried out analyses. DA and SC wrote the manuscript, with contributions from RH. All authors read and approved the submitted version.

## Conflict of Interest

The authors declare that the research was conducted in the absence of any commercial or financial relationships that could be construed as a potential conflict of interest.

## Footnotes

^1^http://grass.cgs.hku.hk/limx/kgg/

^2^http://gn2.genenetwork.org

^3^www.gtexportal.org

## Supplementary Material

FIGURE S1 Enriched Directed Acyclic Graph (DAG) taken from Webgestalt (Zhang et al., 2005; Wang et al., 2013, 2017) showing enriched Gene Ontology (GO) Biological Process (BP) categories for genes coexpressing with *APBB1IP* in human whole blood, taken from GTEXv5. Categories shown in red are enriched in the input dataset, whereas uncolored categories are non-enriched parent-categories. Each box lists the name of the GO category, the number of genes in that category and the significance of enrichment (*p*-value).

FIGURE S2 Enriched Directed Acyclic Graph (DAG) taken from Webgestalt (Zhang et al., 2005; Wang et al., 2013, 2017) showing enriched Gene Ontology (GO) Biological Process (BP) categories for genes coexpressing with *APBB1IP* in human cerebellum, taken from GTEXv5. Categories shown in red are enriched in the input dataset, whereas uncolored categories are non-enriched parent-categories. Each box lists the name of the GO category, the number of genes in that category and the significance of enrichment (*p*-value).

FIGURE S3 Enriched Directed Acyclic Graph (DAG) taken from Webgestalt (Zhang et al., 2005; Wang et al., 2013, 2017) showing enriched Gene Ontology (GO) Biological Process (BP) categories for genes coexpressing with *APBB1IP* in GeneFriends. Categories shown in red are enriched in the input dataset, whereas uncolored categories are non-enriched parent-categories. Each box lists the name of the GO category, the number of genes in that category and the significance of enrichment (*p*-value).

FIGURE S4 Enriched Directed Acyclic Graph (DAG) taken from Webgestalt (Zhang et al., 2005; Wang et al., 2013, 2017) showing enriched Gene Ontology (GO) Biological Process (BP) categories for genes coexpressing with *Apbb1ip* in GeneFriends. Categories shown in red are enriched in the input dataset, whereas uncolored categories are non-enriched parent-categories. Each box lists the name of the GO category, the number of genes in that category and the significance of enrichment (*p*-value).

FIGURE S5 Enriched Directed Acyclic Graph (DAG) taken from Webgestalt (Zhang et al., 2005; Wang et al., 2013, 2017) showing enriched Gene Ontology (GO) Biological Process (BP) categories for genes coexpressing with the *Apbb1ip* in midbrain, from GeneNetwork. Categories shown in red are enriched in the input dataset, whereas uncolored categories are non-enriched parent-categories. Each box lists the name of the GO category, the number of genes in that category and the significance of enrichment (*p*-value).

FIGURE S6 Enriched Directed Acyclic Graph (DAG) taken from Webgestalt (Zhang et al., 2005; Wang et al., 2013, 2017) showing enriched Gene Ontology (GO) Biological Process (BP) categories for genes coexpressing with the *Apbb1ip* eigengene in gastrointestinal tract, from GeneNetwork. Categories shown in red are enriched in the input dataset, whereas uncolored categories are non-enriched parent-categories. Each box lists the name of the GO category, the number of genes in that category and the significance of enrichment (*p*-value).

FIGURE S7 Enriched Directed Acyclic Graph (DAG) taken from Webgestalt (Zhang et al., 2005; Wang et al., 2013, 2017) showing enriched Gene Ontology (GO) Biological Process (BP) categories for genes coexpressing with the *Apbb1ip* eigengene in hypothalamus, from GeneNetwork. Categories shown in red are enriched in the input dataset, whereas uncolored categories are non-enriched parent-categories. Each box lists the name of the GO category, the number of genes in that category and the significance of enrichment (*p*-value).

FIGURE S8 Enriched Directed Acyclic Graph (DAG) taken from Webgestalt (Zhang et al., 2005; Wang et al., 2013, 2017) showing enriched Gene Ontology (GO) Biological Process (BP) categories for genes coexpressing with the *Apbb1ip* eigengene in pituitary gland, from GeneNetwork. Categories shown in red are enriched in the input dataset, whereas uncolored categories are non-enriched parent-categories. Each box lists the name of the GO category, the number of genes in that category and the significance of enrichment (*p*-value).

FIGURE S9 Enriched Directed Acyclic Graph (DAG) taken from Webgestalt (Zhang et al., 2005; Wang et al., 2013, 2017) showing enriched Gene Ontology (GO) Biological Process (BP) categories for genes coexpressing with the *Apbb1ip* eigengene in spleen, from GeneNetwork. Categories shown in red are enriched in the input dataset, whereas uncolored categories are non-enriched parent-categories. Each box lists the name of the GO category, the number of genes in that category and the significance of enrichment (*p*-value).

TABLE S1 Genes with strong, significant (*r* > 0.5 or < −0.5, *p* < 0.05), coexpressing with APBB1IP in human whole blood, taken from GTEXv5. The table shows the record ID (ENSEMBL gene ID), the gene ID (ENTREZ gene ID), the homologene ID, the gene symbol, the gene name, the location of the gene, the mean expression of the gene, the sample *r* (Pearson’s correlation), number of cases, the significance of the sample *r*, the literature correlation (similarity of words used to describe the gene and its function, using latent semantic indexing), and the tissue correlation (correlation between the two samples across tissue types). More details available at GeneNetwork.

TABLE S2 Reactome pathways enriched in genes that are coexpressed with APBB1IP in human whole blood. Gene expression data taken from GTEXv5, enrichment analysis carried out using Webgestalt (Zhang et al., 2005; Wang et al., 2013, 2017). Reactome pathway IDs, the name of the pathway, the number of genes in the pathway, and the false discovery rate (FDR) are shown.

TABLE S3 DrugBank drug targets enriched in genes that are coexpressed with APBB1IP in human whole blood. Gene expression data taken from GTEXv5, enrichment analysis carried out using Webgestalt (Zhang et al., 2005; Wang et al., 2013, 2017). DrugBank IDs, the name of the drug, the number of genes that are targets for the drug, and the false discovery rate (FDR) are shown.

TABLE S4 Expression of APBB1IP in the developing human brain. Prenatal LMD Microarray data was taken from BrainSpan for 516 brain structures at postconception days 15, 16 and 21, using three different probes (Miller et al., 2014).

TABLE S5 Genes with strong, significant (*r* > 0.5 or < −0.5, *p* < 0.05), coexpressing with APBB1IP in human cerebellum, taken from GTEXv5. The table shows the record ID (ENSEMBL gene ID), the gene ID (ENTREZ gene ID), the homologene ID, the gene symbol, the gene name, the location of the gene, the mean expression of the gene, the sample *r* (Pearson’s correlation), number of cases, the significance of the sample r, the literature correlation (similarity of words used to describe the gene and its function, using latent semantic indexing), and the tissue correlation (correlation between the two samples across tissue types). More details available at (GeneNetwork).

TABLE S6 Wikipathways enriched in genes which are coexpressed with APBB1IP in the human cerebellum. Gene expression data taken from GTEXv5, enrichment analysis carried out using Webgestalt (Zhang et al., 2005; Wang et al., 2013, 2017). Wikipathway IDs, the name of the pathway, the number of genes in the pathway, and the false discovery rate (FDR) are shown.

TABLE S7 Genes commonly coexpressed with APBB1IP in human as determined by GeneFriends. This table shows genes that commonly coexpress with *APBB1IP*, as identified by GeneFriends. This coexpression is independent of tissue or treatment. Entrez gene ID, gene symbol and coexpression value with *APBB1IP* are given for each of the genes.

TABLE S8 Genes commonly coexpressed with Apbb1ip in the mouse as determined by GeneFriends. This table shows genes that commonly coexpress with *Apbb1ip*, as identified by GeneFriends. This coexpression is independent of tissue or treatment. Entrez gene ID, gene symbol and coexpression value with *Apbb1ip* are given for each of the genes.

TABLE S9 The top 10,000 probes correlated with expression of Apbb1ip (probe ID 1443145_at) in T-helper cells of the BXD family (GN319; Sloan et al., 2016). The table shows, the probe ID, Mouse gene’s Entrez gene ID, the homologous human gene’s Entrez gene ID, the mouse gene symbol and location in the mouse genome.

TABLE S10 The top 10,000 probes correlated with expression of Apbb1ip (probe ID 1443145_at) in T-regulatory cells of the BXD family (GN122; Sloan et al., 2016). The table shows, the probe ID, Mouse gene’s Entrez gene ID, the homologous human gene’s Entrez gene ID, the mouse gene symbol and location in the mouse genome.

TABLE S11 The top 5,000 probes correlated with expression of Apbb1ip (probe ID A_55_P1961009) in the midbrain of the BXD family (GN381; Sloan et al., 2016). The table shows, the probe ID, Mouse gene’s Entrez gene ID, the homologous human gene’s Entrez gene ID, the mouse gene symbol and location in the mouse genome.

TABLE S12 The top 15,000 probes correlated with the expression of Apbb1ip eigengene in the gastrointestinal tract of the BXD family. The table shows, the probe ID, Mouse gene’s Entrez gene ID, the homologous human gene’s Entrez gene ID, the mouse gene symbol and location in the mouse genome.

TABLE S13 KEGG pathways enriched in genes that are coexpressed with Apbb1ip in mouse hypothalamus. Gene expression data are taken from GeneNetwork (GN159; Sloan et al., 2016), enrichment analysis carried out using Webgestalt (Zhang et al., 2005; Wang et al., 2013, 2017). KEGG pathway IDs, the name of the pathway, the number of genes in the pathway, and the false discovery rate (FDR) are shown.

TABLE S14 The top 16,000 probes correlated with expression of Apbb1ip eigengene in the pituitary of the BXD family (GN335; Sloan et al., 2016). The table shows, the probe ID, Mouse gene’s Entrez gene ID, the homologous human gene’s Entrez gene ID, the mouse gene symbol and location in the mouse genome.

TABLE S15 KEGG pathways enriched in genes that are coexpressed with Apbb1ip in mouse pituitary. Gene expression data are taken from GeneNetwork (GN335; Sloan et al., 2016). enrichment analysis carried out using Webgestalt (Zhang et al., 2005; Wang et al., 2013, 2017). KEGG pathway IDs, the name of the pathway, the number of genes in the pathway, and the false discovery rate (FDR) are shown.

TABLE S16 The top 16,000 probes correlated with expression of Apbb1ip eigengene in the spleen of the BXD family (GN227; Sloan et al., 2016). The table shows, the probe ID, Mouse gene’s Entrez gene ID, the homologous human gene’s Entrez gene ID, the mouse gene symbol and location in the mouse genome.

TABLE S17 The top 16,000 probes correlated with expression of Apbb1ip (probe ID ILM4560441) in erythroid cells of the BXD family (GN150; Sloan et al., 2016). The table shows, the probe ID, Mouse gene’s Entrez gene ID, the homologous human gene’s Entrez gene ID, the mouse gene symbol and location in the mouse genome.
