# Supplementary figures and images for: A Cross-Species Systems Genetics Analysis Links APBB1IP as a Candidate for Schizophrenia and Prepulse Inhibition
Source: Front Behav Neurosci. 2019 Dec 10;13:266. doi: 10.3389/fnbeh.2019.00266 (PMC6914690; doi:10.3389/fnbeh.2019.00266)

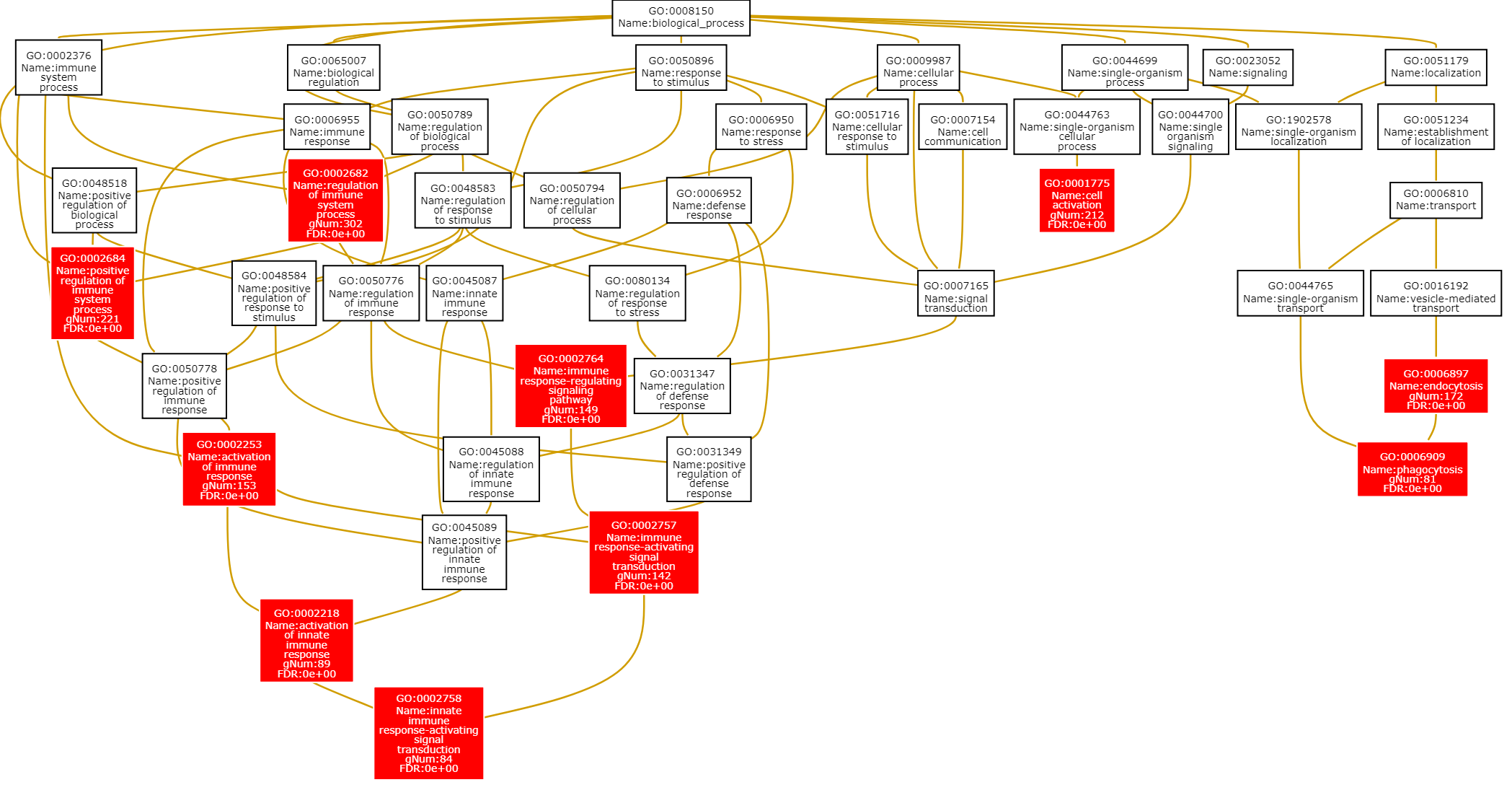

Supplement: FIGURE S1 — Enriched Directed Acyclic Graph (DAG) taken from Webgestalt (Zhang et al., 2005; Wang et al., 2013, 2017) showing enriched Gene Ontology (GO) Biological Process (BP) categories for genes coexpressing with APBB1IP in human whole blood, taken from GTEXv5. Categories shown in red are enriched in the input dataset, whereas uncolored categories are non-enriched parent-categories. Each box lists the name of the GO category, the number of genes in that category and the significance of enrichment (p-value). [file Data_Sheet_1.ZIP › SupplementaryFiles/Supplementary_Figure_1.png]

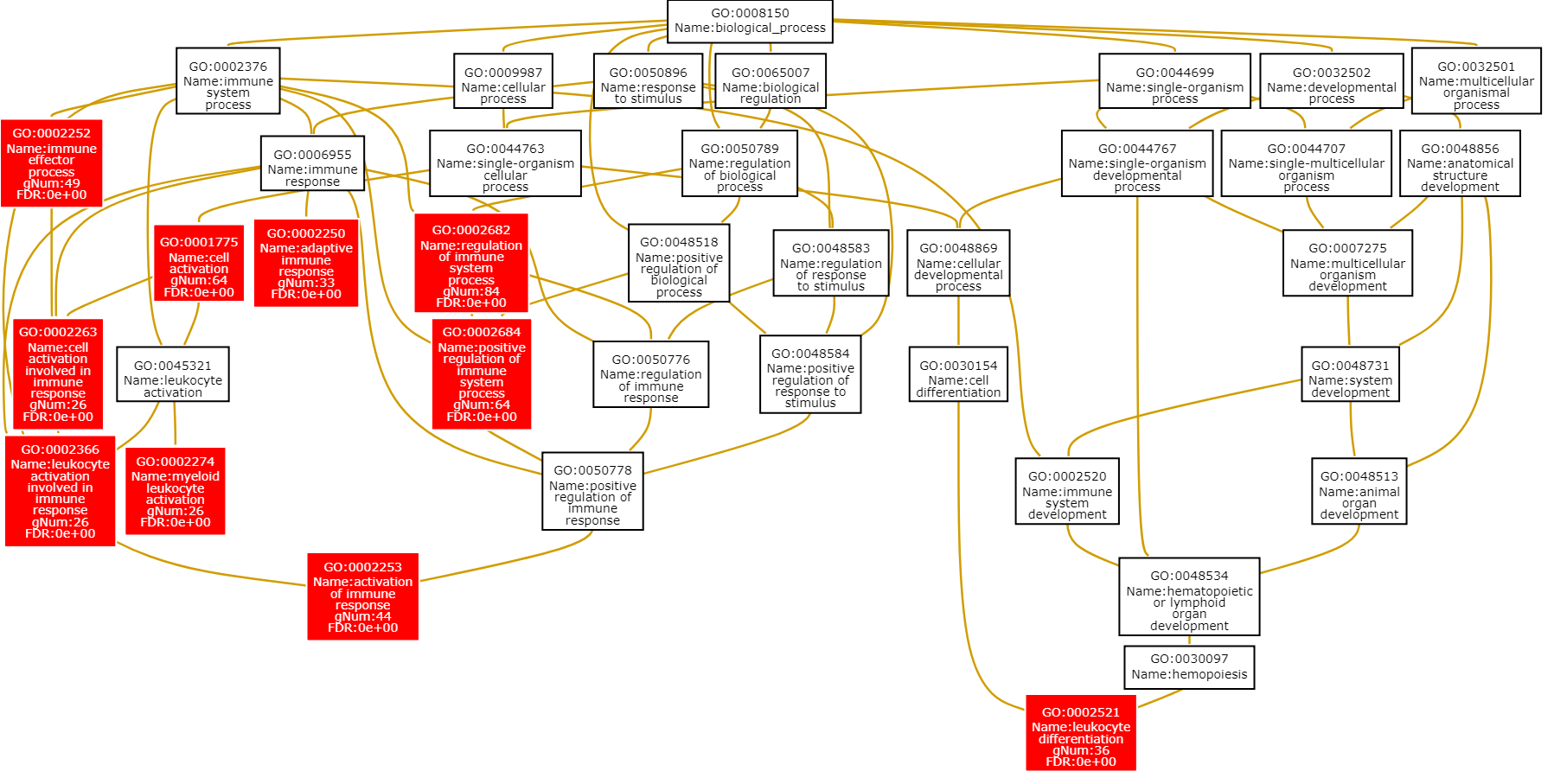

Supplement: FIGURE S1 — Enriched Directed Acyclic Graph (DAG) taken from Webgestalt (Zhang et al., 2005; Wang et al., 2013, 2017) showing enriched Gene Ontology (GO) Biological Process (BP) categories for genes coexpressing with APBB1IP in human whole blood, taken from GTEXv5. Categories shown in red are enriched in the input dataset, whereas uncolored categories are non-enriched parent-categories. Each box lists the name of the GO category, the number of genes in that category and the significance of enrichment (p-value). [file Data_Sheet_1.ZIP › SupplementaryFiles/Supplementary_Figure_2.png]

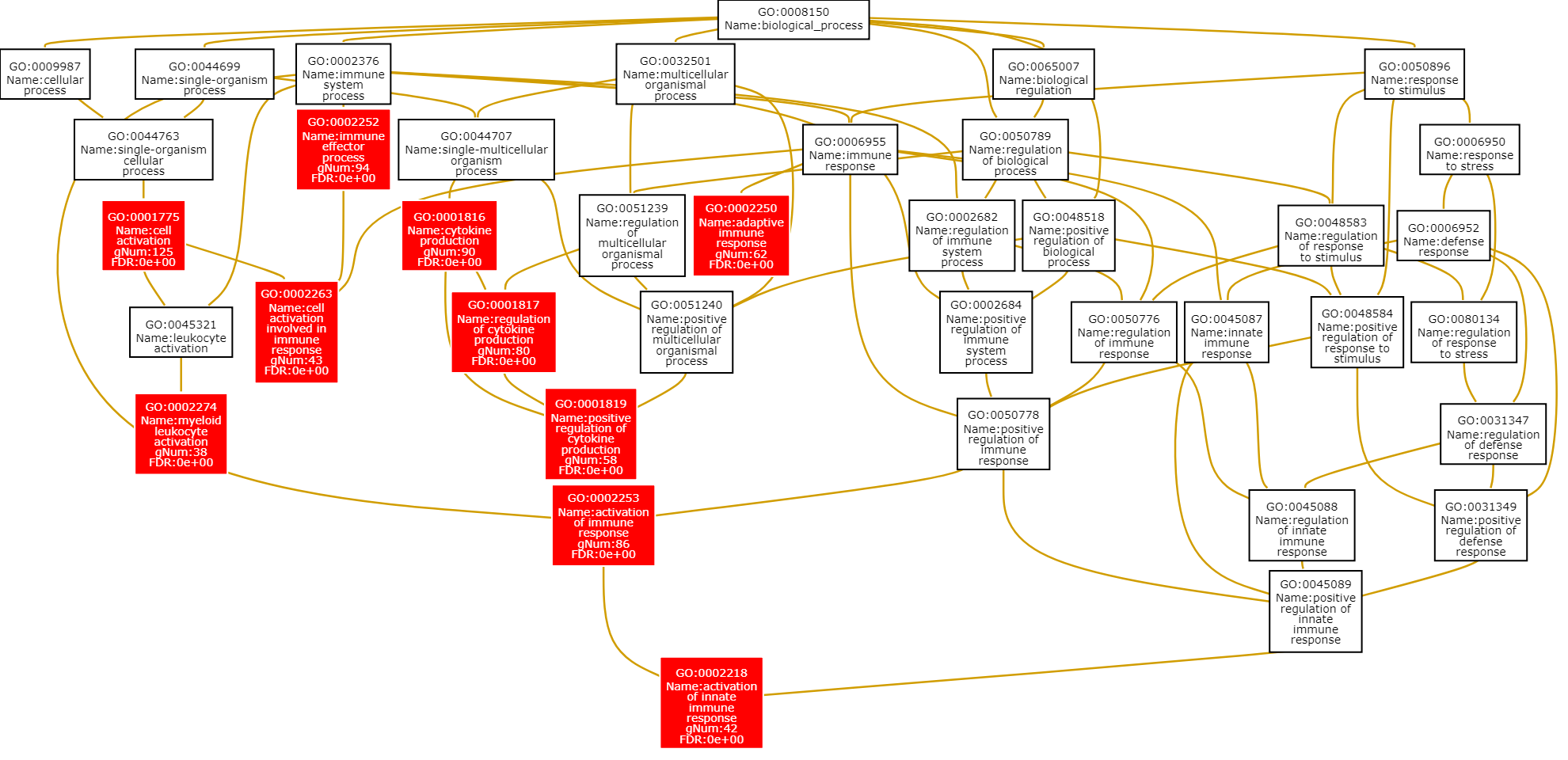

Supplement: FIGURE S1 — Enriched Directed Acyclic Graph (DAG) taken from Webgestalt (Zhang et al., 2005; Wang et al., 2013, 2017) showing enriched Gene Ontology (GO) Biological Process (BP) categories for genes coexpressing with APBB1IP in human whole blood, taken from GTEXv5. Categories shown in red are enriched in the input dataset, whereas uncolored categories are non-enriched parent-categories. Each box lists the name of the GO category, the number of genes in that category and the significance of enrichment (p-value). [file Data_Sheet_1.ZIP › SupplementaryFiles/Supplementary_Figure_3.png]

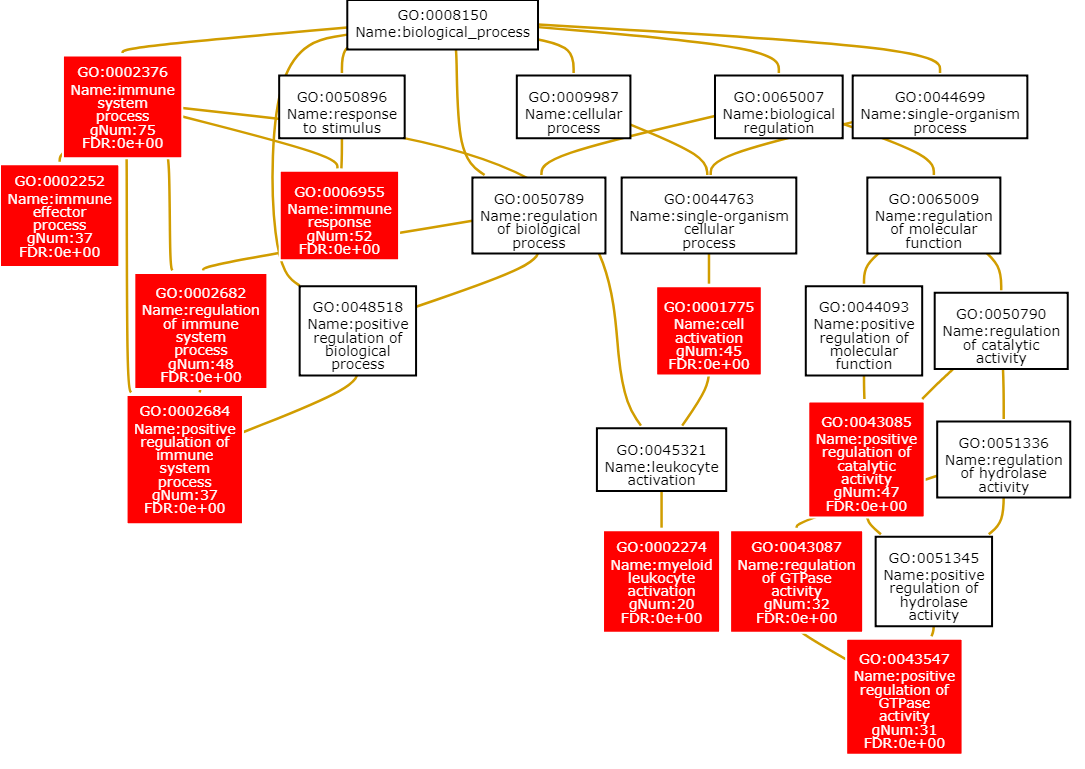

Supplement: FIGURE S1 — Enriched Directed Acyclic Graph (DAG) taken from Webgestalt (Zhang et al., 2005; Wang et al., 2013, 2017) showing enriched Gene Ontology (GO) Biological Process (BP) categories for genes coexpressing with APBB1IP in human whole blood, taken from GTEXv5. Categories shown in red are enriched in the input dataset, whereas uncolored categories are non-enriched parent-categories. Each box lists the name of the GO category, the number of genes in that category and the significance of enrichment (p-value). [file Data_Sheet_1.ZIP › SupplementaryFiles/Supplementary_Figure_4.png]

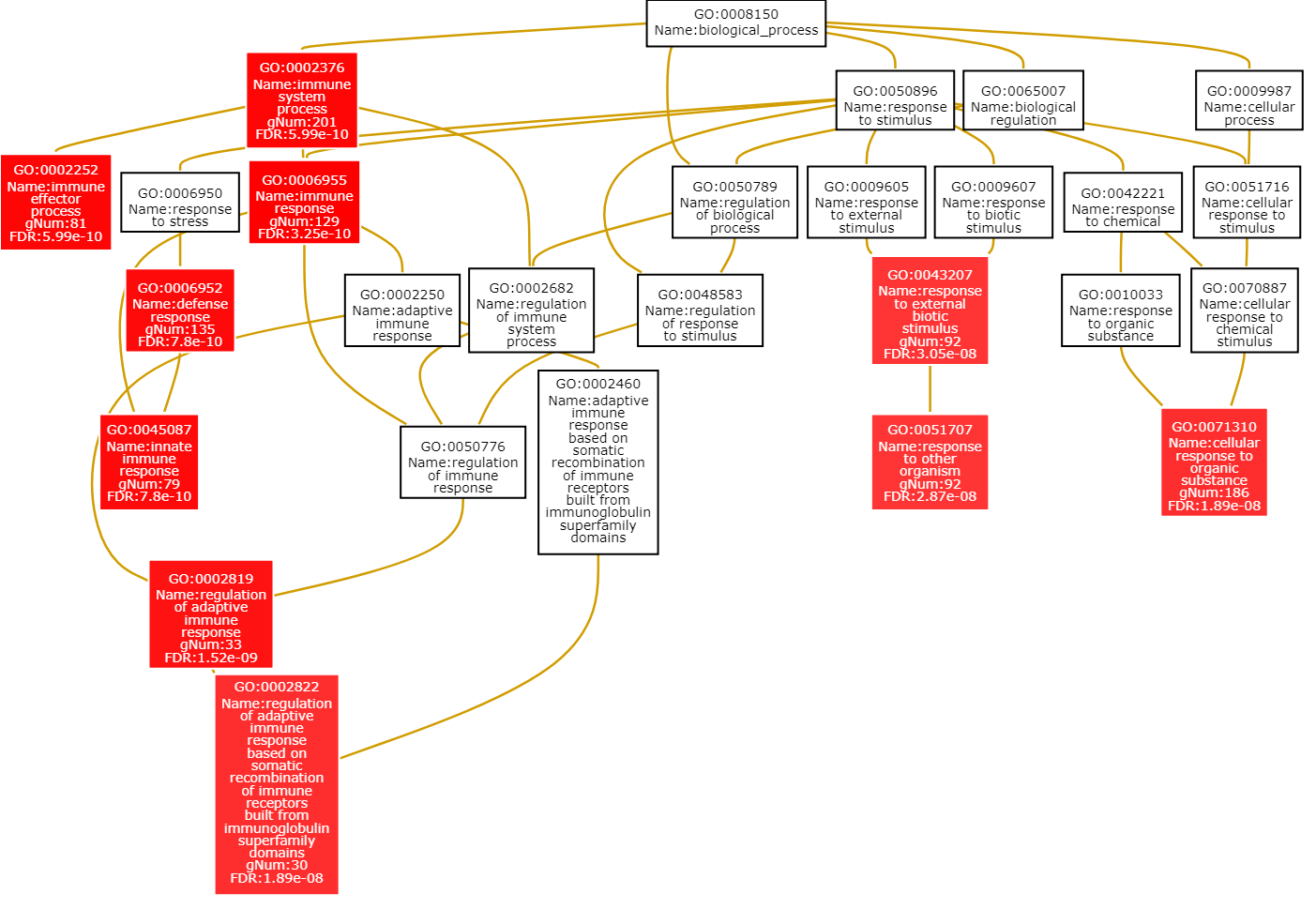

Supplement: FIGURE S1 — Enriched Directed Acyclic Graph (DAG) taken from Webgestalt (Zhang et al., 2005; Wang et al., 2013, 2017) showing enriched Gene Ontology (GO) Biological Process (BP) categories for genes coexpressing with APBB1IP in human whole blood, taken from GTEXv5. Categories shown in red are enriched in the input dataset, whereas uncolored categories are non-enriched parent-categories. Each box lists the name of the GO category, the number of genes in that category and the significance of enrichment (p-value). [file Data_Sheet_1.ZIP › SupplementaryFiles/Supplementary_Figure_5.png]

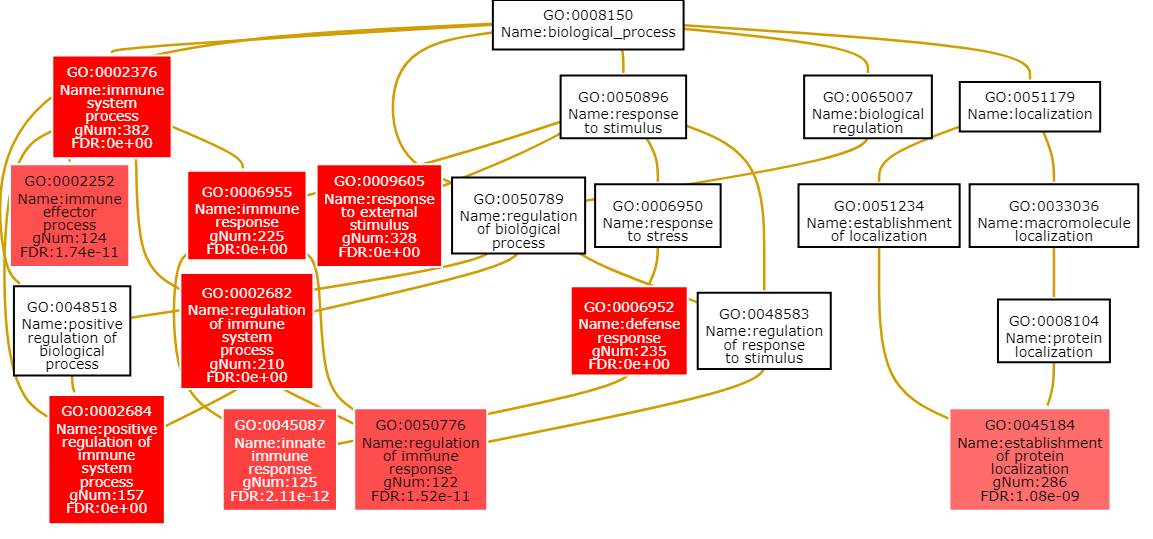

Supplement: FIGURE S1 — Enriched Directed Acyclic Graph (DAG) taken from Webgestalt (Zhang et al., 2005; Wang et al., 2013, 2017) showing enriched Gene Ontology (GO) Biological Process (BP) categories for genes coexpressing with APBB1IP in human whole blood, taken from GTEXv5. Categories shown in red are enriched in the input dataset, whereas uncolored categories are non-enriched parent-categories. Each box lists the name of the GO category, the number of genes in that category and the significance of enrichment (p-value). [file Data_Sheet_1.ZIP › SupplementaryFiles/Supplementary_Figure_6.png]

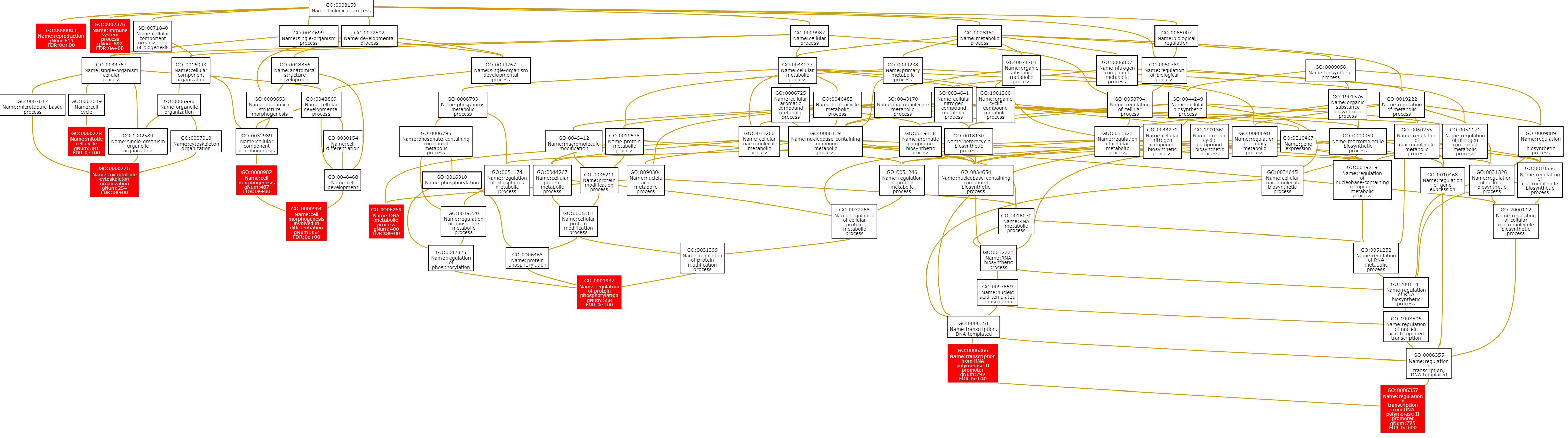

Supplement: FIGURE S1 — Enriched Directed Acyclic Graph (DAG) taken from Webgestalt (Zhang et al., 2005; Wang et al., 2013, 2017) showing enriched Gene Ontology (GO) Biological Process (BP) categories for genes coexpressing with APBB1IP in human whole blood, taken from GTEXv5. Categories shown in red are enriched in the input dataset, whereas uncolored categories are non-enriched parent-categories. Each box lists the name of the GO category, the number of genes in that category and the significance of enrichment (p-value). [file Data_Sheet_1.ZIP › SupplementaryFiles/Supplementary_Figure_7.png]

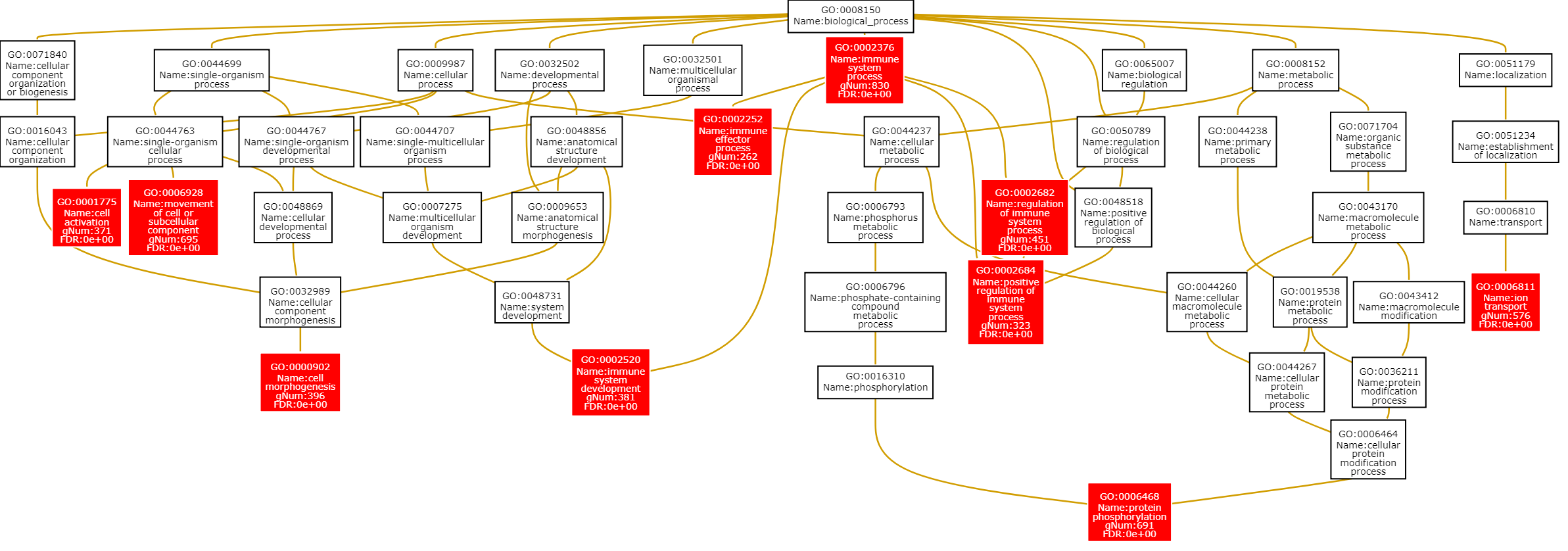

Supplement: FIGURE S1 — Enriched Directed Acyclic Graph (DAG) taken from Webgestalt (Zhang et al., 2005; Wang et al., 2013, 2017) showing enriched Gene Ontology (GO) Biological Process (BP) categories for genes coexpressing with APBB1IP in human whole blood, taken from GTEXv5. Categories shown in red are enriched in the input dataset, whereas uncolored categories are non-enriched parent-categories. Each box lists the name of the GO category, the number of genes in that category and the significance of enrichment (p-value). [file Data_Sheet_1.ZIP › SupplementaryFiles/Supplementary_Figure_8.png]

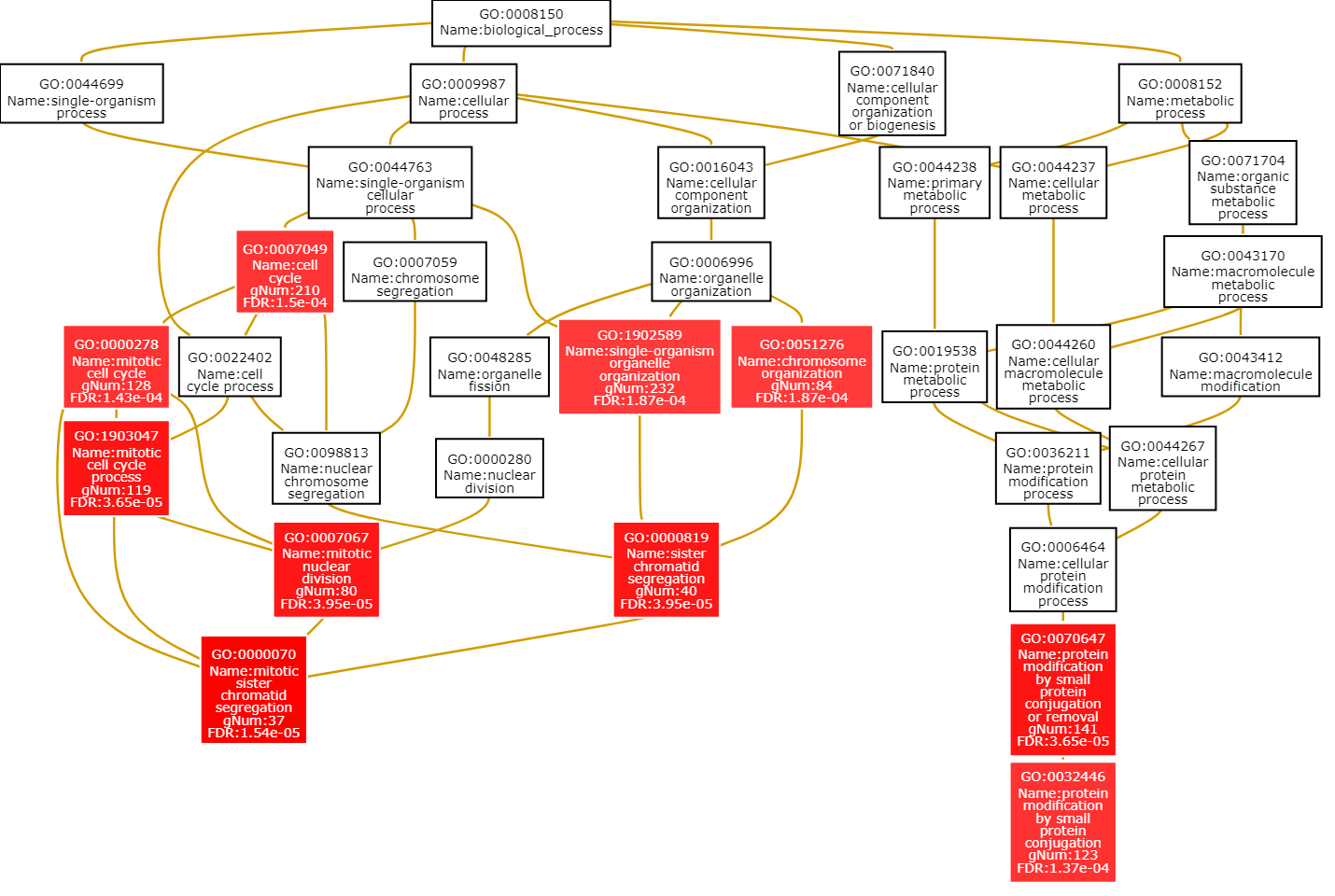

Supplement: FIGURE S1 — Enriched Directed Acyclic Graph (DAG) taken from Webgestalt (Zhang et al., 2005; Wang et al., 2013, 2017) showing enriched Gene Ontology (GO) Biological Process (BP) categories for genes coexpressing with APBB1IP in human whole blood, taken from GTEXv5. Categories shown in red are enriched in the input dataset, whereas uncolored categories are non-enriched parent-categories. Each box lists the name of the GO category, the number of genes in that category and the significance of enrichment (p-value). [file Data_Sheet_1.ZIP › SupplementaryFiles/Supplementary_Figure_9.png]
